# Guillain-Barré Syndrome Clinical Characteristics and Outcomes Among U.S. Active Component Service Members, 2014–2022

**Published:** 2026-02-24

**Authors:** Emily J. Elliott, Shauna L. Stahlman, Nora Watson, Cole Denkensohn, Kaye Sedarsky

**Affiliations:** Naval Aerospace Medical Institute, Pensacola, FL: LCDR Elliott; Epidemiology and Analysis Branch, Armed Forces Health Surveillance Division, Public Health Directorate, Defense Health Agency, Silver Spring, MD: Dr. Stahlman; Walter Reed National Military Medical Center, Bethesda, MD: Dr. Watson, Dr. Sedarsky; Landstuhl Regional Medical Center, Landstuhl, Germany: MAJ Denkensohn

## Abstract

An examination of Guillain-Barré Syndrome (GBS) cases among U.S. active component service members from 2014 through 2022 revealed an incidence rate of 1.6 cases per 100,000 person-years. Individuals younger than age 20 years and those in basic training exhibited higher incidence. The type of antecedent event, either illness or immunization, was not associated with higher disability ratings at long-term follow-up. The analysis also quantified morbidity among service members with GBS, finding that 28.0% of cases had a subsequent chronic pain diagnosis, and 28.7% of cases were referred to the medical evaluation board. The need for neuropathic pain medication during the acute phase predicted poorer long-term functional outcomes. Furthermore, electrodiagnostic evidence of axonal or mixed nerve damage correlated with greater disability after 1 year. Although basic trainees had higher incidence, their long-term morbidity was comparable to other groups. These findings underscore the considerable impact that GBS can have on affected military personnel and identify factors associated with long-term complications.

**What are the new findings?**

There were 1.6 cases of Guillain-Barré syndrome per 100,000 person years among active component U.S. service members from 2014 through 2022. There was no association between persistent disability and associated antecedent event (e.g., infection or immunization). Many patients experienced incomplete recovery, with 28.7% resulting in medical board referrals. Persistent disability was independently associated with chronic pain diagnosis.

**What is the impact on readiness and force health protection?**

Despite the low incidence rate of the disorder, approximately 29% of U.S. service member GBS cases experienced incomplete recovery that required medical board referral. Service members appear to be at a higher risk for GBS during initial recruit basic training, potentially due to increased exposure to infections and immunization requirements at accession.

Guillain-Barré syndrome (GBS) is an acute immune-mediated polyradiculoneuropathy. GBS stems from an autoimmune response related to an antecedent illness, immunization, or other immune reaction causing damage to myelin (acute inflammatory demyelinating polyneuropathy, or AIDP) or axons (acute motor axonal neuropathy, or AMAN) of the peripheral nerves and ganglia. AIDP is the predominant variant seen in North America. ^1^

GBS occurs with an overall world-wide incidence rate (IR) of 0.6–4.0 cases per 100,000 people per year with higher rates reported in North America, 2.2–4.2 cases per 100,000. ^2 - 8^ One study from 2009 found a slightly higher incidence of GBS in the active duty U.S. military population compared to the general population. ^5^ It is more common in men and can affect all age groups. ^1^

Mortality due to GBS varies in reported studies, from 2% to 10%, with predictors including advanced age, mechanical ventilation, and cardiopulmonary complications. ^1 - 4 , 6^ Morbidity with severe disability can be seen in upwards of 20% of patients, with predictors including advanced age, mechanical ventilation, preceding diarrheal illness, and high-grade disability in the acute phase. ^1 - 4^ Pain is a common symptom upon presentation and can persist long term, significantly affecting quality of life. ^9^

Classic clinical presentation of GBS manifests as a progressive ascending muscle weakness with decreased or absent deep tendon reflexes. ^6^ Patients also present with sensory symptoms, ataxia, lower back pain, and cranial nerve involvement that range in severity. ^6^ Autonomic dysfunction is also common and can be fatal. ^6^ Variants include pure motor, pure sensory, Miller Fisher, pharyngeal-brachial, and paraparetic. ^6^

There are no specific biomarkers associated with GBS. Diagnosis of GBS is typically based on a thorough history and clinical examination. Certain diagnostic tools may support diagnosis, including cerebrospinal fluid (CSF) analysis, serum antibody testing, magnetic resonance imaging (MRI), and electrodiagnostic studies.

The disease timeline is typically monophasic, with progression over 2 weeks and symptom nadir (i.e., most critically ill point) around 4 weeks after onset. ^6^ Severity is variable, and up to one-fourth of cases require mechanical ventiliation. ^6 , 8^ Close monitoring and early initiation of intravenous immunoglobulins (IVIG) or plasma exchange (PLEX) is essential for accelerating recovery. ^7^ Uncommonly, acute clinical presentation of GBS can herald another neurological disorder, such as chronic inflammatory demyelinating polyneuropathy (CIDP) or neurological presentation of other systemic diseases such as lupus or infection.

Antecedent respiratory or gastrointestinal illness can be identified in up to three-fourths of patients presenting with GBS. ^1 , 8 , 10^ *Campylobacter jejuni* is the most common prior infection, with 30% of cases in 1 study demonstrating serological evidence of the infection. ^10^ Other infectious etiologies include *Mycoplasma pneumonia* , cytomegalovirus, Epstein-Barr virus, hepatitis E virus, Zika, dengue, and influenza. ^1 , 8 , 10^ Asymptomatic infections have also been detected by serological testing, which may suggest higher rates of antecedent illness. ^10^

The risk of immunization-related GBS was originally based on the 1976 swine influenza vaccine, but studies investigating influenza immunization after 1976 had mixed results, with most showing no causal relationship. ^11^ Low, but increased risk of GBS following adenovirus-vectored COVID-19 vaccines was lower than the risk identified with the 1976 influenza vaccine. ^12^ The same study also found reduced risk of GBS with the messenger RNA (mRNA) COVID-19 vaccines. ^12^ The U.S. Centers for Disease Control and Prevention (CDC)'s Advisory Committee on Immunization Practices (ACIP) states that GBS is not a precaution for future immunizations, unless it occurred within 6 weeks of receiving a tetanus-toxoid-containing vaccine or an influenza vaccine. ^13^

Immunizations are administered upon accession into military service, unless a service member provides prior documentation of prior immunization or serological testing showing presence of antibodies. ^14^ Immunization administration upon military accession is recommended before or at the beginning of basic training, to help mitigate risk of contagious disease in close quarters environments. ^14^ Additional immunizations such as yellow fever, Japanese encephalitis, and rabies may be required depending upon travel or area of operation requirements. ^14^ COVID-19 immunization was mandated for all military members in August 2021; however, the mandate was rescinded in January 2023. Immunizations identified by the CDC of potential concern are influenza and tetanus vaccines, however, other vaccines have also been implicated, including yellow fever and rabies. ^15 , 16^

A previous military population study, of matched case-control design, evaluated the association between GBS and acute gastrointestinal infections and deployment from 1999 through 2007. ^5^ That 2009 study identified a slightly higher incidence in the military cohort compared to the general population, but it was limited by retrospective database review without medical record review. ^5^

The objective of the current study was to describe the incidence, clinical characteristics (including antecedent illness or immunization), clinical course, and electrodiagnostic findings of U.S. active component service members (ACSMs) with clinically confirmed GBS from 2014 through 2022. Due to the timeline chosen for data extraction, it includes 2 years of COVID-19 immunization in addition to yearly influenza immunization. An updated, comprehensive understanding of the clinical characteristics of GBS, its disease course, and their readiness implications will supply health care providers with knowledge that can aid patient education, improve prognostication discussions, and potentially assuage apprehensions about immunizations in relation to GBS risk.

## Methods

Potential cases of GBS were identified as those with documentation of an International Classification of Diseases, 9th or 10th Revision, Clinical Modification (ICD-9-CM / ICD-10-CM) code (357.0 or G61.0, respectively) in an inpatient or outpatient medical encounter from January 1, 2014 through December 31, 2022 among ACSMs in the U.S. Army, Navy, Marine Corps, Coast Guard, Air Force, or Space Force. The data came from medical records maintained in the Defense Medical Surveillance System (DMSS) that the authors obtained from the Armed Forces Health Surveillance Division (AFHSD) in 2023. DMSS ICD-9-CM / ICD-10-CM code queries included diagnostic positions of 4 digits for outpatient records and 9 digits for inpatient records. The records examined included those from military hospitals and clinics as well as civil ian medical facilities if reimbursement was sought through the Military Health System (MHS). The 2014 start date was chosen to capture treatment and prescription data through DMSS. The Walter Reed National Military Medical Center determined this project to be human subject research exempt from institutional board review.

A list of 401 potential cases identified in DMSS was sent to the primary investigator's research team of neurologists and neurology residents for individual record review **(Figure)** . Cases were excluded during individual chart review when the diagnosis code was entered with no other supporting information to confirm diagnosis, or the diagnosis was revised during the acute treatment period. Cases were also excluded if the diagnosis code referenced childhood or prior history of GBS before January 2014.

**FIGURE 1.**
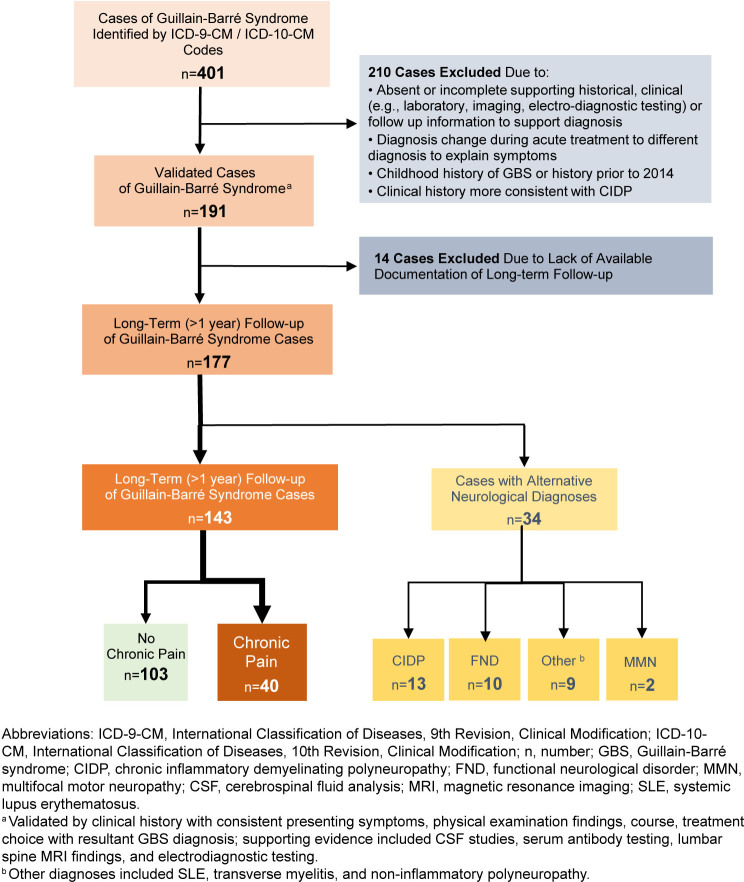
Subject Identification Flow Chart for Guillain-Barré Syndrome Cases, U.S. Active Component Service Members, 2014–2022

Following individual chart reviews, 191 cases were identified as acute presentations of GBS. Cases were validated based on a culmination of consistent clinical history, symptoms upon patient presentation, physical examination findings, and treatment choice consistent with GBS diagnosis. Supporting diagnostic evidence including CSF studies, serum antibody testing, lumbar spine MRI findings, and electrodiagnostic testing were also reviewed to aid case validation. Data collected for the 191 identified cases included patient demographics, clinical information, electrodiagnostic testing data, and related case outcomes.

The acute phase of GBS was considered as the time from initial clinical evaluation to either end of acute treatment course, final hospitalization, or acute rehabilitation discharge. Clinical information collected in the acute phase included presence of antecedent illness or prior immunization, timeline of symptom onset, diagnostics (e.g., laboratory, imaging, electrodiagnostic data), primary treatments, pain treatment, hospital care and complications, and disability rating, using the Modified Rankin Scale (MRS), at the most critically ill point (i.e., nadir) of acute presentation. Antecedent illness information was obtained from clinical history and review of clinical notes 30 days prior to presentation, for indications of acute illness appointments or infection treatments. Immunization information was obtained from clinical histories, reviews of medical chart immunization records, and reviews of clinical notes indicating immunization appointments within 30 days preceding patient presentation. Any immunization within 30 days was recorded according to type of immunization and date administered.

Clinical information collected after the acute phase included additional electrodiagnostic study data, time to recovery, chronic pain diagnosis, and long-term follow-up MRS. Electrodiagnostic testing results were classified as normal, demyelinating, or mixed / axonal. Demyelinating cases had isolated demyelinating features that could include prolonged or absent F-waves, prolonged distal latencies, or slowed conduction velocities. Axonal or mixed cases had either axonal features alone or axonal and demyelinating features. Axonal features could include low amplitude action potentials or spontaneous activity on electromyography.

Time to recovery was assessed by patient report of recovery, date returned to full duty, and medical evaluation board (MEB) referrals. MEB information was compared and validated with MEB information provided by the DMSS. Chronic pain diagnosis based on presence of ICD-9-CM or ICD-10-CM code (338.2 or G89) or documentation of chronic pain within the clinical note and was dichotomized as present or not.

During chart reviews, chronic pain diagnosis was included if it could be related to GBS diagnosis in documentation of chronic pain present since acute phase. A diagnosis of chronic pain was included if pain was a new symptom at time of GBS diagnosis and continued at 1 year followup or beyond. Cases were not included in the chronic pain category if chronic pain diagnosis was clearly attributable to another injury. Chronic pain diagnosis was independently confirmed by the primary investigator.

MRS determination was based on reviews of neurologists' interpretations of clinical disability reports from histories and documented physical examination findings. If multiple clinical encounters were available after 1 year of follow-up, then the encounter closest to 1 year after the original diagnosis was used to determine MRS. The MRS scale is a disability scale graded from 0 to 6, with 0 indicating no symptoms present, 1 indicating minimal symptoms but ability to complete all usual activities, 2 indicating slight disability but able to perform daily activities without assistance, 3 indicating moderate disability requiring help and unable to walk alone without assistance, 4 indicating moderate severe disability requiring assistance for own bodily needs, 5 indicating severe disability unable to attend own body needs without constant assistance, and 6 indicating death. ^17^

All reviewers were trained on proper application of the MRS scale, and a small sampling of cases was provided to the reviewers prior to initiation of review to aid with inter-rater reliability. The primary investigator confirmed complementary assessments with all reviewers of the sample cases provided. A data collection sheet for the acute phase collected medical research council sum score, ventilation requirements, and medical complications during hospitalization, which aided determination of nadir MRS scores. The primary investigator independently confirmed all MRS scores with the data collection sheet and independent review.

The list of confirmed cases was returned to AFHSD for IR calculation. AFHSD used longitudinal personnel data in the DMSS to calculate the rate of clinically confirmed GBS per 100,000 person-years (p-yrs) of active component service. Person-time was censored at the incident diagnosis date. Person time for recruit basic training was identified using a standard AFHSD algorithm based on time in service, assigned military installation, branch of service, and other factors.

Case characteristics were described and compared by MRS and chronic pain diagnosis as outcomes after 1 year follow-up using Wilcoxon 2-sample tests and Chi-square or Fisher's exact tests. To evaluate potential independent associations of case characteristics with each outcome, multivariable logistic regression models estimated adjusted odds ratios associated with several characteristics selected by the research team based both on their potential clinical relevance and association with the outcome in unadjusted analyses. Further adjustment was avoided to guard against model overfitting and to conform to the traditional minimum number of events per predictor (> 10) in logistic regression. Low variance inflation factors in each model indicated that multi-collinearity was not a concern.

The dependent variables were MRS outcome and chronic pain diagnosis at 1 year or greater follow-up. MRS was defined as either 0 (asymptomatic) or greater than 0 (minimally symptomatic to severe disability). Independent variables included use of neuropathic pain treatment in the acute treatment phase, presence of pain in the acute phase, and significant disability (MRS > 3) during the acute phase. A dichotomized MRS definition of greater than or equal to 3 was based both on observed elevated frequencies of each outcome in this stratum relative to lower MRS scores and by the small cell sizes in several MRS strata, which prevented further evaluation of MRS as an ordinal score in multivariable models. These analyses were performed using R (version 4.0.5). ^18^

## Results

The DMSS database identified 401 potential GBS cases by ICD-9-CM / ICD-10-CM codes alone **(Figure)** . After individual chart reviews, 191 cases were identified as acute GBS. An IR of 1.6 confirmed GBS cases per 100,000 p-yrs was calculated for ACSMs from 2014 through 2022 **(Table 1)** . Male service members had a slightly higher rate than female service members (1.7 and 1.0 cases per 100,000 p-yrs, respectively). There was a higher IR in those younger than age 20 years, at 3.6 cases per 100,000 p-yrs, and those in basic training recruit status (11.5 cases per 100,000 p-yrs).

**TABLE 1.** Incidence Rate ^a^ of Guillain-Barré Syndrome, U.S. Active Component Service Members, 2014–2022

Characteristics	No.	Rate
Total	191	1.6
Sex		
Male	171	1.7
Female	20	1.0
Age, *y*		
<20	29	3.6
20–24	55	1.5
25–29	33	1.2
30–34	32	1.7
35–39	19	1.4
40–44	16	2.2
45+	7	1.6
Race and ethnicity		
White, non-Hispanic	76	2.4
Black, non-Hispanic	21	2.3
Hispanic	30	1.6
Other	15	1.5
Unknown	49	1.0
Basic training recruit status		
Yes	28	11.5
No	163	1.4

Abbreviations: No., number; *y* , years.

a Per 100,000 person-years.

An isolated antecedent illness within 30 days of diagnosis was noted in 94 of 191 (49.2%) cases **(Table 2)** . Median time from illness start to diagnosis date was 11 days. Isolated immunization within 30 days of the date of diagnosis was seen in 28 (14.7%) cases. Median time from immunization to date of diagnosis was 15 days. Approximately one-fourth of cases, 46 of 191 (24.1%), had both an antecedent illness and immunization within 30 days of the preceding syndrome presentation.

**TABLE 2.** Clinical Characteristics of U.S. Active Component Service Members with Guillain-Barré Syndrome, 2014–2022

Clinical Feature	No.	% ^a^
Total	191	100
Antecedent clinical event		
Preceding illness only	94	49.2
Preceding immunization only	28	14.7
Both preceding illness and immunization	46	24.1
No preceding illness or immunization	23	12.0
Pain present, acute phase		
Yes	141	73.8
No	50	26.2
Modified Rankin Scale nadir		
MRS 0 (no symptoms)	0	0.0
MRS 1 (no significant disability)	40	20.9
MRS 2 (slight disability)	71	37.2
MRS 3 (moderate disability)	33	17.3
MRS 4 (moderately severe disability)	29	15.2
MRS 5 (severe disability)	16	8.4
Unknown	2	1.0
Hospitalization		
Yes	177	92.7
No	14	7.3
Median duration	7 days (2–54)	
Mechanical ventilation required		
Yes	20	10.4
No	171	89.6
Primary treatment		
IVIG	155	81.2
None	11	5.8
IVIG / PLEX	11	5.8
PLEX	7	3.7
Steroids	7	3.7
Neuropathic pain treatment		
Yes	92	48.2
No	99	51.8
Electrodiagnostic testing completed		
Yes	108	56.5
No	83	43.5
Results of electrodiagnostic testing		
Not completed	83	43.5
Demyelinating	53	27.7
Axonal, mixed	28	14.7
Normal	27	14.1

Abbreviations: No., number; MRS, Modified Rankin Scale; IVIG, intravenous immunoglobulins; PLEX, plasma exchange.

a Row percentage calculated from total cases defined with clinical feature.

The most reported symptoms were upper respiratory illness or influenza-like illness, which were seen in 101 (52.8%) cases. COVID infection was only noted in 4 (2.1%) cases. Influenza was the most common immunization received within the preceding 30 days (11.5%). COVID immunization had been received in 9 (4.7%) cases. Among the 28 basic training recruits, 9 of the 28 (32.1%) had a prior illness only, 7 of the 28 (25%) had a previous immunization only, and 12 of the 28 (42.8%) had both a concurrent preceding illness and immunization (data not shown).

Pain was reported during initial patient presentation in 141 of 191 (73.8%) cases **(Table 2)** . Over three-fourths of cases demonstrated no significant disability to moderate disability at the most critical point of initial presentation, ranging from MRS 1 in 40 cases (20.9%), MRS 2 in 71 cases (37.2%), and MRS 3 in 33 cases (17.3%). Almost one-fourth of cases had moderate to severe disability at nadir, with MRS 4 (n=29, 15.2%) or 5 (n=16, 8.4%).

All but 14 patients were hospitalized for monitoring and management **(Table 2)** . The median duration of hospitalization was 7 days (range 2–54 days). Eleven patients received no documented treatment. Most patients received IVIG as primary treatment (n=155, 81.2%). Eleven patients received IVIG in combination with PLEX. Seven patients received PLEX alone, and 7 received steroids alone for primary treatment. Neuropathic pain medication was given to 48.2% (n=92) of patients.

Over half of cases (n=108, 56.5%) received electrodiagnostic testing. Among the 108 cases with electrodiagnostic testing, results were interpreted as normal in 27 cases; evidence of isolated demyelinating features was noted in 53 cases; and 28 cases had axonal or mixed findings **(Table 2)** . Serial electrodiagnostic studies were completed in 45 of 108 (41.6%) cases (data not shown). Follow-up studies were normal in 15 of 45 (33.3%) cases, with median follow-up testing at 51 days (range 7–896 days). Demyelinating features were present in 20 of 45 cases (44.4%), with median follow-up testing at 152 days (range 6–1,063 days). Axonal or mixed features were observed in 10 of 45 cases (22.2%) with median follow-up testing at 122 days (range 19–1,191 days). Five cases had more than 2 serial electrodiagnostic tests completed (4 of 5 were axonal, range 35–1,088 days). One case with primary axonal damage had persistent fibrillation potentials and positive waves more than 1 year after original diagnosis (data not shown).

Following the initial treatment course, 36 patients had recurrent or persistent symptoms without interval improvement after initial hospitalization (data not shown). One case (0.5%) was believed to be recurrence of GBS, 3.5 years after the initial episode. Five cases experienced symptom recrudescence within 90 days, attributed to the initial disease presentation, that were subsequently treated with second rounds of IVIG. Five cases had recurrent symptoms more than 90 days later, without other objective evidence of recurrence, with 1 receiving IVIG treatment again 6 years later.

Long-term follow-up more than 1 year after initial diagnosis was available for 177 cases. Five cases had no clear improvement after acute presentation and continued to report persistent symptoms at long-term follow-up. Following the acute phase, alternative diagnoses (i.e., other than GBS) were made or suspected in 34 cases. Among those 34 cases, CIDP was the ultimate diagnosis in 13 cases; multifocal motor neuropathy was diagnosed in 2 cases; and other neurological disorders were suspected in 19 cases, including 10 cases of functional neurological disorder. Median time to symptom recurrence for CIDP was 33 days, and 130 days for all others with polyphasic presentations.

After excluding the 34 cases with possible alternative long-term diagnoses, there were 143 cases of GBS with more than 1 year of follow-up **(Table 3)** . MRS was extracted from clinical encounters at least 1 year after original diagnosis. During follow-up after 1 year, 73 of 143 (51.0%) cases had returned to baseline, with MRS 0. The outcomes of long-term follow-up for all other cases were distributed from MRS 1 in 46 (32.2%) cases, MRS 2 in 17 (11.9%) cases, MRS 3 in 3 (2.1%) cases, MRS 4 in 3 (2.1%) cases, and MRS 6 in 1 case with death unrelated to GBS.

**TABLE 3.** Outcomes of Long-Term Follow-up of More Than One Year, U.S. Active Component Service Members with Guillain-Barré Syndrome, 2014–2022

		Cases With No Alternative Diagnoses		Total Cases	
Total follow-up cases, after 1 year	143 ^a^		177 ^b^	
Modified Rankin Scale, after 1 year	No.	%	No.	%
MRS 0 (no symptoms)	73	51.0	76	43.0
MRS 1 (no significant disability)	46	32.2	62	35.0
MRS 2 (slight disability)	17	11.9	29	16.4
MRS 3 (moderate disability)	3	2.1	5	2.8
MRS 4 (moderately severe disability)	3	2.1	4	2.3
MRS 5 (severe disability)	0	0.0	0	0.0
MRS 6 (deceased)	1 ^c^	0.7	1	0.5
Return to full duty ^d^				
Yes	93	65.0	100	56.5
No	50	35.0	77	43.5
Average time	5 mo.		5 mo.	
Median time	4 mo.		4 mo.	
Chronic pain diagnosis				
Yes	40	28.0	53	30.0
No	103	73.0	124	70.0

Abbreviations: No., number; MRS, Modified Rankin Scale; mo., months; CIDP, chronic inflammatory demyelinating polyneuropathy; FND, functional neurologic disorder; GBS, Guillain-Barré syndrome.

a Long-term follow-up cases excluding those with other diagnoses: CIDP, FND, other neurological diagnosis.

b 14 of original 191 cases had no long-term follow-up documentation.

c Did not die from GBS.

d Did not return to full duty includes medical board, permanent profile, separated before return to full duty documented.

The average time for return to duty in those with full recovery was 5 months (median 4 months, range 0.5–40 months). Type of antecedent clinical event and recruit status were not associated with higher MRS at 1 year follow-up ( *p* =0.182 and *p* =0.077, respectively) **(Table 4a)** . Patients with axonal or mixed electrodiagnostic results were more likely to have MRS greater than 0 at 1 year versus patients with demyelinating or normal electrodiagnostic testing ( *p* <0.001) **(Table 4a)** .

**TABLE 4a.** Demographic and Clinical Characteristic Associated with Long-Term ^a^ Modified Rankin Scale Outcomes

		No Symptoms Indicated per Modified Rankin Scale		Symptoms Indicated per Modified Rankin Scale		*p* -value
Total, *n*	73 ^b^		70 ^b^			
		No.	%	No.	%	
Recruit status					0.077
No	61	83.6	66	94.3	
Yes	12	16.4	4	5.7	
Antecedent event					0.182
Illness	33	45.2	41	58.6	
Immunization	9	12.3	9	12.9	
Both	24	32.9	12	17.1	
Neither	7	9.59	8	11.4	
Pain present, acute phase					0.021
No	26	35.6	12	17.1	
Yes	47	64.4	58	82.9	
Neuropathic pain treatment					<0.001
No	57	78.1	23	32.9	
Yes	16	21.9	47	67.1	
Modified Rankin Scale nadir, acute phase					0.027
>3	24	32.9	38	54.3	
0–1	21	28.8	13	18.5	
2	28	38.4	19	27.1	
Chronic pain diagnosis					<0.001
No	72	98.6	31	44.3	
Yes	1	1.39	39	55.7	
Results of electrodiagnostic testing ^c^					<0.001
Total, *n*	33 ^c^		41 ^c^		
Demyelinating	21	63.6	18	43.9	
Axonal, mixed	1	3.0	18	43.9	
Normal	11	33.3	5	12.2	

Abbreviations: MRS, Modified Rankin Scale; *n* , number; No., number; CIDP, chronic inflammatory demyelinating polyneuropathy; FND, functional neurologic disorder.

a Greater than 1 year.

b Long-term follow-up cases, excluding those with other diagnoses: CIDP, FND, other neurological diagnosis.

c Long-term follow-up cases with electro-diagnostic testing results, excluding cases with other diagnoses: CIDP, FND, other neurological diagnosis.

Chronic pain associated with GBS diagnosis was seen in 40 (28.0%) of the 143 cases with no other alternative diagnosis at long-term follow-up **(Table 3)** . Chronic pain diagnoses were seen more commonly in patients with MRS greater than 0 at 1 year ( *p* <0.001) **(Table 4a)** . Among the 143 cases at long-term follow-up, MEB was initiated for 41 (28.7%) cases, and 93 cases (65.0%) returned to duty. Twenty-six of the 41 referred for MEB had a chronic pain diagnosis (data not shown).

Of the 40 patients with chronic pain diagnoses, 33 (82.5%) initially required neuropathic pain treatment ( *p* <0.001) **(Table 4b)** . In a multi-variable logistic regression model of relevant clinical characteristics as predictors of MRS outcome and chronic pain diagnosis at long-term follow-up, neuropathic pain treatment was associated with greater risk of MRS greater than 0 (OR 6.8; 95% CI 2.8, 17.7; *p* <0.001) and resultant chronic pain diagnosis (OR 7.9; 95% CI 2.7, 28.0; *p* <0.001) independent of reported pain and MRS at nadir **(Table 5)** .

**TABLE 4b.** Demographic and Clinical Characteristic Associations with Long-Term Follow-up Presence of Chronic Pain Diagnosis

Chronic Pain Diagnosis	No		Yes		*p* -value
Total, *n*	103 ^a^		40 ^a^		
		No.	%	No.		%
Recruit status					0.236
No	89	86.4	38	95.0	
Yes	14	13.6	2	5.0	
Modified Rankin Scale nadir, acute phase					0.080
> 3	39	37.9	23	59.0	
0–1	27	26.2	6	15.4	
2	37	35.9	10	25.6	
Pain present, acute phase					<0.001
No	37	35.9	1	2.5	
Yes	66	64.1	39	97.5	
Neuropathic pain treatment					<0.001
No	73	70.6	7	17.5	
Yes	30	29.4	33	82.5	
Results of electrodiagnostic testing ^b^					0.035
Total, *n*	50 ^b^		23 ^b^		
Axonal, mixed	9	18.0	10	43.5	
Demyelinating	27	54.0	11	47.8	
Normal	14	28.0	2	8.7	

Abbreviations: No., number; *n* , number; CIDP, chronic inflammatory demyelinating polyneuropathy; FND, functional neurologic disorder.

a Long-term follow-up cases, excluding those with other diagnosis: CIDP, FND, other neurological diagnosis.

b Long-term follow up cases with electrodiagnostic results, excluding cases with other diagnosis: CIDP, FND, other neurological diagnosis.

**TABLE 5.** Multivariable Logistic Regression Model of Clinical Characteristics with Morbidity Outcomes at Long-Term Follow-up (after 1 year)

Characteristic	Odds Ratio	95% CI Lower Limit	95% CI Upper Limit	*p* -value
Modified Rankin Scale, after 1 year				
Neuropathic pain treatment ^a^				
Yes	6.8	2.8	17.7	<0.001
No	Reference			
Pain reported, acute phase ^a^				
Yes	0.8	0.3	2.3	0.72
No	Reference			
Modified Rankin Scale nadir, > 3 ^a^ , acute phase				
Yes	1.6	0.8	3.5	0.022
No	Reference			
Chronic pain diagnosis, after 1 year				
Neuropathic pain treatment ^a^				
Yes	7.9	2.7	28.0	<0.001
No	Reference			
Pain reported, acute phase ^a^				
Yes	5.5	0.8	107.7	0.13
No	Reference			
Modified Rankin Scale nadir, > 3 ^a^				
Yes	1.4	0.55	3.3	0.5
No	Reference			

Abbreviation: CI, confidence interval; >, greater than; MRS, Modified Rankin Scale.

a Neuropathic pain treatment defined as identified as present (“Yes” vs. “No”); pain present during acute phase (“Yes” vs. “No”); MRS at nadir greater than or equal to (>=) 3 for significant disability (“Yes” vs. “No”).

## Discussion

This study provides an update on clinical characteristics and outcomes in GBS among a large military cohort. GBS shares an overall similar IR in the U.S. military population when compared to reported rates globally ^1 , 4 , 7 - 9^ but has a lower incidence when compared to directly to other North American and European cohorts (1.9 to 4.2 per 100,000 p-yrs). ^2 , 7^ GBS severity is variable, but there were no fatalities attributable to GBS in this cohort. This lack of mortality may be related to a lower rate of mechanical ventilation (10.4% vs. up to 23% in other studies ^3 , 8^ ) and an overall healthier and younger active duty military population.

Results of electrodiagnostic testing can be helpful in predicting long-term MRS outcomes, but only slightly more than half of cases in this study had electrodiagnostic testing available for review. This study did not identify a clear role for serial electrodiagnostic testing. Serial testing can be beneficial, however, if the diagnosis is in question or the patient has persistent or recurring symptoms. It is notable that 1 axonal case had persistent fibrillation potentials and positive waves in serial electrodiagnostic testing more than 1 year after initial diagnosis; this appears to have captured the natural course of the disease rather than representing a second pathology.

This study quantified morbidity associated with GBS in U.S. ACSMs, as seen in the 28.0% of cases associated with a subsequent chronic pain diagnosis, and in 28.7% of cases referred to the MEB. The 2009 military study reported 20% of service members with continued medical visits related to GBS 1 year post-diagnosis, and other studies report approximately 20% with long-term disability. ^4 , 5^ There were similar rates of reported pain in the acute period (~70%) and long-term follow-up period (~25%) compared to a recent civilian cohort. ^9^ The need for treatment with neuropathic pain medication could be an early indicator for morbidity, as it was independently associated with MRS of greater than 0 at followup. This may represent an additional and relatively early clinical feature to consider when determining overall prognosis. Additionally, it may provide an impetus to consider earlier or more aggressive treatment in certain cases. Future prospective studies could provide further clarification.

Determination of the type of GBS, axonal versus demyelinating, can be helpful with understanding associated acute and chronic pain, prognostication, and differentiating GBS from other mimickers. ^4 , 6 , 9^ A recent study highlighted that acute pain may be more pronounced in axonal variants while chronic pain may be associated with demyelinating, however, this was in a cohort of Asian subjects, who typically have higher axonal rates. ^9^ If there is diagnostic or prognostic uncertainty after the acute phase, electrodiagnostic testing should be pursued.

There was no significant association between type of preceding event (e.g., infection or immunization) and long-term morbidity. Those in basic training recruit status did, however, have a higher incidence when compared to other groups. The recruit population typically receives multiple immunizations upon arrival, and they are also housed in close quarters, increasing potential infection transmissibility. This was reflected in this analysis, as most recruits had a preceding illness or immunization within 30 days of symptom onset. This study was not designed to determine direct causal relationships with immunization.

The main limitation of this study is its retrospective design. While these findings can provide insights, correlation cannot be established. Utilization of medical encounter data is both limited by accuracy of documentation and subject to reporting bias. Cases may be under-reported due to reliance on appropriate ICD-9-CM / ICD-10-CM code placement. Risk factors cannot be assessed with this study design. Antecedent illness and prior immunization data can be incomplete if not documented in clinical notes, or if service members received immunizations out of network without records in MHS medical charts. Evaluating morbidity by using an MRS greater than 0 may over-estimate significant disability.

This study provides an important update on GBS in the active component population of the U.S. military for MHS clinicians and may help guide future research. Given the increased incidence during the recruit training period in this study, it is prudent for health care providers working with populations such as military recruits to consider referral to an appropriate level of care if suspicion of GBS arises, in addition to ensuring appropriate immunization counseling and addressing elements to help mitigate close quarters disease transmissibility. Despite the increased incidence of GBS in recruits, no evidence supported a higher risk of long-term morbidity in the U.S. active component population.

## References

[1] van den Berg B , Walgaard C , Drenthen J , et al . Guillain-Barré syndrome: pathogenesis, diagnosis, treatment and prognosis . Nat Rev Neurol . 2014 ; 10 ( 8 ): 469 - 482 . doi: 10.1038/nrneurol.2014.121 25023340

[2] Bragazzi NL , Kolahi AA , Nejadghaderi SA , et al . Global, regional, and national burden of Guillain–Barré syndrome and its underlying causes from 1990 to 2019 . J Neuroinflammation . 2021 ; 18 ( 1 ): 264 . doi: 10.1186/s12974-021-02319-4 34763713 PMC8581128

[3] Alshekhlee A , Hussain Z , Sultan B , Katirji B . Guillain-Barré syndrome: incidence and mortality rates in US hospitals . Neurology . 2008 ; 70 ( 18 ): 1608 - 1613 . doi: 10.1212/01.wnl.0000310983.38724.d4 18443311

[4] Chiò A , Cocito D , Leone M , et al . Guillain-Barré syndrome: a prospective, population-based incidence and outcome survey . Neurology . 2003 ; 60 ( 7 ): 1146 - 1150 . doi: 10.1212/01.wnl.0000055091.96905.d0 12682322

[5] Nelson L , Gormley R , Riddle MS , Tribble DR , Porter CK . The epidemiology of Guillain-Barré syndrome in U.S. military personnel: a case-control study . BMC Res Notes . 2009 ; 26 ( 2 ): 171 . doi: 10.1186/1756-0500-2-171 PMC2739856 19709434

[6] Willison HJ , Jacobs BC , van Doorn PA . Guillain-Barré syndrome . Lancet . 2016 ; 388 ( 10045 ): 717 - 727 . doi: 10.1016/s0140-6736(16)00339-1 26948435

[7] Shahrizaila N , Lehmann HC , Kuwabara S . Guillain-Barré syndrome . Lancet . 2021 ; 397 ( 10280 ): 1214 - 1228 . doi: 10.1016/s0140-6736(21)00517-1 33647239

[8] Finsterer J . Triggers of Guillain-Barré syndrome: *Campylobacter jejuni* predominates . Int J Mol Sci . 2022 ; 23 ( 22 ): 14222 . doi: 10.3390/ijms232214222 36430700 PMC9696744

[9] Papri N , Mohammed A , Rahman MM , et al . Pain determinants and quality of life in Guillain-Barré syndrome: a prospective cohort study . BMJ Neurol Open . 2024 ; 6 ( 2 ): e000925 . doi: 10.1136/bmjno-2024-000925 PMC11647308 39687605

[10] Leonhard SE , et al , IGOS Consortium . An international perspective on preceding infections in Guillain-Barré syndrome: the IGOS-1000 cohort . Neurology . 2022 ; 99 ( 12 ): e1299 - e1313 . doi: 10.1212/wnl.0000000000200885 35981895

[11] Salmon DA , Proschan M , Forshee R , et al . Association between Guillain-Barré syndrome and influenza A (H1N1) 2009 monovalent inactivated vaccines in the USA: a meta-analysis . Lancet . 2013 ; 381 ( 9876 ): 1461 - 1468 . doi: 10.1016/s01406736(12)62189-8 23498095

[12] Censi S , Bisaccia G , Gallina S , Tomassini V , Uncini A . Guillain-Barré syndrome and COVID-19 vaccination: a systematic review and meta-analysis . J Neurology . 2024 ; 271 ( 3 ): 1063 - 1071 . doi: 10.1007/s00415-024-12186-7 PMC10896967 38233678

[13] Eiffert SR , Stürmer T , Thorpe CT , et al . Vaccine patterns among patients diagnosed with Guillain-Barré syndrome and matched counterparts in a Medicare supplemental population, 2000–2020 . Vaccine . 2023 ; 41 ( 39 ): 5763 - 5768 . doi: 10.1016/j.vaccine.2023.08.014 37573203 PMC10528847

[14] Headquarters, Departments of the Army, the Navy, the Air Force, and the Coast Guard. Army Regulation 40-562, BUMEDINST 6230.15B, AFI 48-110_IP, CG COMDTINST M6230.4G–Medical Services: Immunizations and Chemoprophylaxis for the Prevention of Infectious Diseases. U.S. Dept. of Defense . Oct . 7 , 2013 . Accessed Nov. 19, 2025 . https://www.med.navy.mil/portals/62/documents/nmfa/nmcphc/root/field%20activities/pages/nepmu6/operational%20support/environmental%20health/bumedinst-6230-15b-immunizations-and-chemoprophylaxis.pdf

[15] Staples J , Gershman M , Fischer M , U.S. Centers for Disease Control and Prevention. Yellow fever vaccine: recommendations of the Advisory Committee on Immunization Practices (ACIP) . MMWR Recomm Rep . 2010 ; 59 ( rr-7 ): 1 - 27 . Accessed Nov. 19, 2025 . https://www.cdc.gov/mmwr/pdf/rr/rr5907.pdf 20671663

[16] Hemachudha T , Griffin D , Chen W , Johnson R . Immunologic studies of rabies vaccination-induced Guillain-Barré syndrome . Neurology . 1988 ; 38 ( 3 ): 375 - 378 . doi: 10.1212/wnl.38.3.375 2450302

[17] Saver JL , Chaisinanunkul N , Campbell BCV , et al , XIth Stroke Treatment Academic Industry Roundtable. Standardized nomenclature for Modified Rankin Scale global disability outcomes: consensus recommendations from Stroke Therapy Academic Industry Roundtable XI . Stroke . 2021 ; 52 ( 9 ): 3054 - 3062 . doi: 10.1161/strokeaha.121.034480 34320814

[18] The R Foundation . The R Project for Statistical Computing . Accessed Nov. 19, 2025 . https://www.r-project.org

